# Standardization of ^68^Ge/^68^Ga Using Three Liquid Scintillation Counting Based Methods

**DOI:** 10.6028/jres.113.020

**Published:** 2008-10-01

**Authors:** B. E. Zimmerman, J. T. Cessna, R. Fitzgerald

**Affiliations:** Ionization Radiation Division, National Institute of Standards and Technology, Gaithersburg, MD 20899

**Keywords:** anticoincidence counting, CIEMAT/NIST method, germanium-68, liquid scintillation counting, positron emitter, standardization, TDCR method

## Abstract

A solution containing ^68^Ge in equilibrium with its daughter, ^68^Ga, has been standardized for the first time at the National Institute of Standards and Technology (NIST) using 3 liquid scintillation-based techniques: live-timed 4π*β* -*γ* anticoincidence (LTAC) counting, the Triple-to-Double Coincidence Ratio (TDCR) method, and ^3^H-standard efficiency tracing with the CIEMAT^1^/NIST (CNET) method. The LTAC technique is much less dependent on level scheme data and model-dependent parameters and was thus able to provide a reference activity concentration value for the master solution with a combined standard uncertainty of about 0.3 %. The other two methods gave activity concentration values with respective differences from the reference value of +1.2 % and −1.5 %, which were still within the experimental uncertainties. Measurements made on the NIST “4π”*γ* secondary standard ionization chamber allowed for the determination of calibration factors for that instrument, allowing future calibrations to be made for ^68^Ge/^68^Ga without the need for a primary measurement. The ability to produce standardized solutions of ^68^Ge presents opportunities for the development of a number of NIST-traceable calibration sources with very low (<1 %) relative standard uncertainties that can be used in diagnostic medical imaging.

## 1. Introduction

The use of Positron Emission Tomography (PET) as a tool for diagnosing diseases, particularly cancer, continues to rise at a rate of about 20 % per year [1], partially due to technological advances that allow for more quantitative data to be collected. The ability to consistently acquire truly quantitative imaging data depends on the use of radioactivity standards traceable to the National Institute of Standards and Technology (NIST).

Currently the most widely used radionuclide in PET imaging is ^18^F. Although NIST has previously standardized ^18^F [2,3], its short half-life (1.83 h) makes it nearly impossible to prepare and distribute Standard Reference Materials to most users. As a potential solution, ^68^Ge, in equilibrium with its daughter, ^68^Ga, has been proposed as a surrogate.

**Fig. 1:**
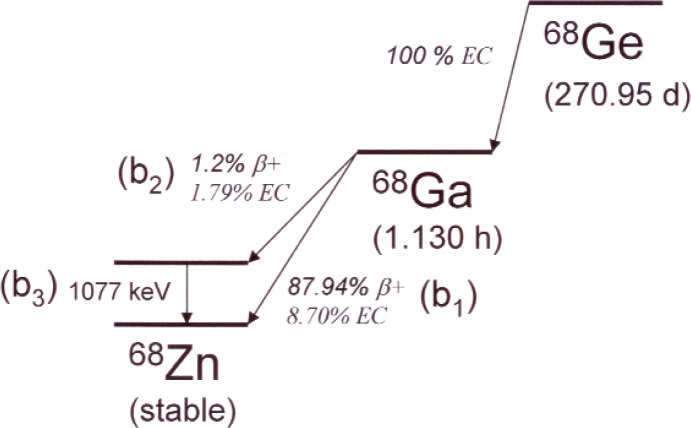
Simplified decay scheme for ^68^Ge-^68^Ga. Data were taken from the DDEP data evaluation [4]. The branching probabilities b_1_, b_2_, and b_3_ refer to the two positron emission and gamma emission probabilities, respectively.

A simplified scheme for the decays of ^68^Ge and its ^68^Ga daughter is shown in Fig. 1. Germanium-68 decays by pure electron capture (EC) to the ground state of ^68^Ga with a half-life of 270.95(16) d [4]. Gallium-68 in turn decays with a half-life of 67.71(9) min by a combination of EC and positron emission primarily to the ground state of ^68^Zn, but also with a branch to an excited state at 1077 keV with a probability of about 3 % and a number of higher excited states with a combined probability of under 0.4 %.

The nature of the decay scheme of ^68^Ge/^68^Ga makes it amenable to a variety of different standardization techniques. In 1994, Schönfeld, et al. [5] reported on the results of measurements made with liquid scintillation (LS) counting using the CIEMAT/NIST ^3^H standard efficiency tracing method (CNET) [6,7], 4π*β* - *γ* coincidence, and a calibrated ionization chamber (IC). The data showed good agreement between all three methods, giving activity concentration values within the respective experimental uncertainties (nominally 1 % relative standard uncertainty).

More recently, Grigorescu, et al. [8], reported on the results of measurements using 4π*β* - *γ* coincidence counting. As with Schönfeld, et al., the coincidence spectrometer consisted of a proportional counter and NaI(Tl) detector for the *β* and *γ* detection channels, respectively. Because this experimental arrangement requires the use of dried sources, corrections for the loss of ^68^Ge due to chemical volatility were necessary in both studies. This effect is reported by Grigorescu to be on the order of 20 % to 26 %. Nonetheless, they were able to obtain a measurement result with about a 1 % relative standard uncertainty.

Liquid scintillation (LS) counting has been the method of choice in our laboratory for the measurement of *β*-emitting radionuclides, primarily due to the high LS detection efficiency and the relative ease of sample preparation. Methods based on LS counting have another advantage in the context of measuring ^68^Ge because the sample is introduced into the cocktail while still in solution, thereby eliminating the need to prepare dry sources. Seeking to take advantage of this, we have measured a single solution of ^68^Ge/^68^Ga using three LS-based methods: live-timed 4π*β* - *γ* anticoincidence (LTAC) using LS as the *β* counting channel, the Triple-to-Double Coincidence Ratio Method (TDCR) [9,10], and CNET [6,7].

## 2. Experimental

All evaluation of measurement uncertainties throughout this work follow accepted conventions used by the NIST Radioactivity Group and are in accordance with those recommended by the principal metrology organizations [11]. All individual uncertainty components are given as estimated experimental standard deviations (or standard deviations of the mean, if appropriate), or quantities assumed to correspond to standard deviations regardless of the method used to evaluate their magnitude. Unless explicitly stated, all uncertainties cited in this paper are “standard uncertainties,” corresponding to one uncertainty interval. One particular exception is the uncertainty reported for the activity concentration of the calibrated ^68^Ge solution, which is given as an “expanded combined standard uncertainty.” In accordance with NIST policy [12], the combined standard uncertainty (calculated by combining the individual uncertainty components in quadrature) is multiplied by a “coverage factor” of *k* = 2 to obtain an “expanded uncertainty” assumed to give an uncertainty interval having a confidence level of 90 % to 95 %.

### 2.1 Initial Solution Preparation

The master solution used in these experiments contained nominally 125 MBq ^68^Ge in 5 mL of 0.5 mol · L^−1^ HCl and was prepared by International Isotopes Idaho, Inc. (Idaho Falls, ID)^2^ using ^68^Ge produced at the 100 MeV Isotope Production Facility at Los Alamos National Laboratory using the ^nat.^Ga(p,2n)^68^Ge reaction.

**Fig. 2:**
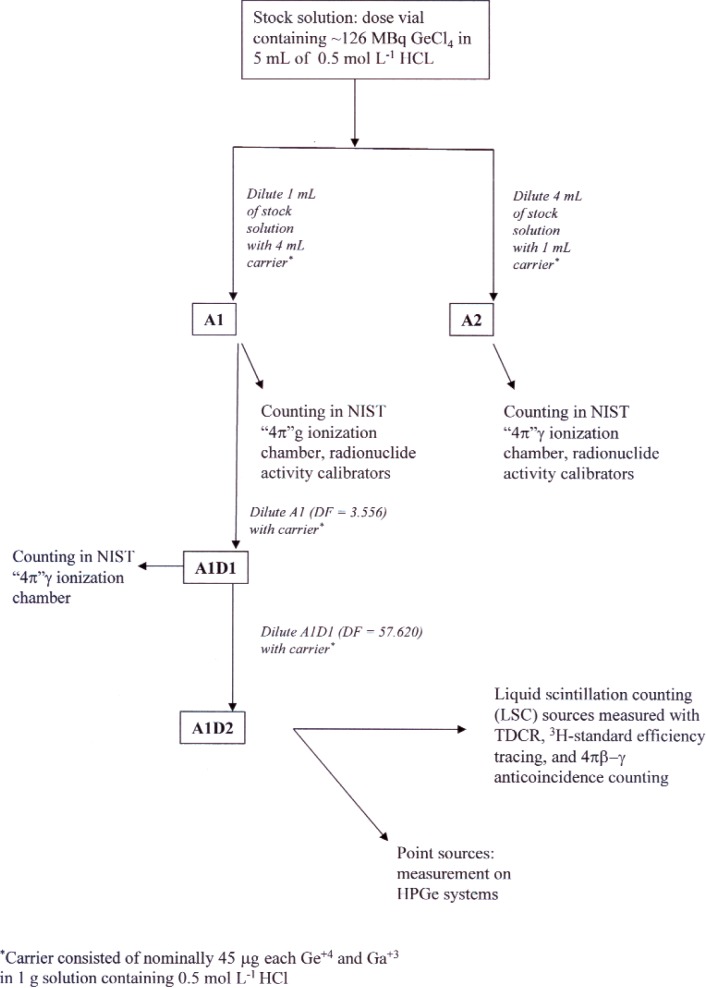
Scheme used for preparation of sources used to calibrate solutions of ^68^GeCl_4_.

A generalized scheme for the preparation of the counting samples is shown in Fig. 2. The first step involved transfer of the master solution out of the shipping vial into a NIST standard 5 mL flame-sealed ampoule while at the same time performing the first of three serial dilutions that would be needed in order to bring the activity level in one of the ampoules down to that suitable for LS counting. The ampoule used for this study, labeled *A1*, was prepared by volumetrically adding 1 mL of the stock solution to an ampoule containing 4 g of gravimetrically added carrier solution having nominally 45 μg each of nonradioactive Ge^+4^ and Ga^+3^ per gram of solution using 0.5 mol · L^−1^ HCl as the solvent. The ampoule was weighed again after the addition of the ^68^Ge to determine the mass of added radioactive solution. Ampoule *A2*, shown in Fig. 2, was held in reserve for future experiments.

Ampoule *A1* was measured in the NIST-maintained radionuclide activity calibrators (“dose calibrators”) and the NIST “4π”*γ* IC [13] to allow for the determination of calibration factors in this specific measurement geometry. The solution in *A1* was then diluted by a factor of about 200 through two serial gravimetric dilutions, giving two additional ampoules, *A1D1* and *A1D2*. As an additional check of the dilution factor between *A1* and *A1D1*, the latter was also measured in the NIST IC.

### 2.2 Liquid Scintillation Source Preparation

All counting sources for the three counting techniques were prepared using solution *A1D2*. A total of 18 LS cocktails containing ^68^Ge were prepared for these studies. For the LTAC experiments, 4 mL of HiSafe-3 (Perkin Elmer, Waltham, MA) or PCS (GE Healthcare Biosciences, Piscataway, NJ) scintillant were added to each of two 3 cm diameter, glass pseudo-hemispheres. Nominally 0.04 g of ^68^Ge solution were gravimetrically added and the hemispheres were sealed using epoxy.

For the TDCR experiments, two cocktails each of HiSafe-3 and PCS were prepared by dispensing 10 mL of the scintillant into four 22 mL borosilicate glass LS vials (two per scintillant), followed by the gravimetric addition of nominally 0.04 g of solution from *A1D2*. Similarly, 10 cocktails were prepared for the CNET experiments (five vials per scintillant). To vary the counting efficiency of the CNET cocktails, between 2 drops and 18 drops of a 10:1 (by volume) dilution of nitromethane in ethanol were added as a quenching agent to the CNET cocktails in addition to the scintillant and radioactive solution. In order to perform the efficiency tracing, a separate set of 10 LS vials having identical composition to the ^68^Ge cocktails were prepared using a dilution of a NIST tritiated water Standard Reference Material 4927F [14] in place of the ^68^Ge. In order to make the ^68^Ge and ^3^H cocktails as close in composition as possible, nominally 1 mL of the Ge^4+^/Ga^3+^ carrier was added to each of the cocktails.

Two background blanks (one for each scintillant) were prepared for the TDCR and LTAC measurements in their appropriate vials. In order to properly mimic the composition of the radioactive cocktails, an equivalent mass of nonradioactive carrier solution was added to each blank. For the CNET measurements, four blanks were prepared so as to have the identical sample compositions of the least- and most-quenched of the radioactive ^68^Ge cocktails. As with the TDCR and LTAC blanks, nonradioactive Ge/Ga carrier was substituted for the ^68^Ge solution.

### 2.3 4π*β* - *γ* Anticoincidence Counting (LTAC)

The system constructed at NIST uses an LS source optically coupled to an appropriate photomultiplier tube for the beta channel and a thallium-doped sodium iodide [NaI(Tl)] detector for the *γ*-ray channel, as described previously [15,16]. The LS-based system is well suited to this case since the source solution does not have to be dried, and therefore, the large (20 % to 26 %) correction for Ge loss reported by Grigorescu [8] is avoided.

The four active sources were each measured for between 2 and 4 cycles and the blank sources up to 3 cycles during the period from 24 April to 5 May 2007. Each counting cycle consisted of measurements at between 8 to 12 threshold levels on the LS detector for between 200 and 1000 seconds. A minimum of 5 · 10^6^ LS and 1 · 10^5^ anti-coincident NaI detector counts were recorded for each non-blank measurement. The LS signal-to-background ratio for the lowest threshold (highest background) data points was about 950:1, while the signal-to-background ratio for the NaI detector was about 350:1. Further systematic tests demonstrated that the background variability during the span of the experiment, the variation of extending dead-time, and the presence or absence of the aluminum absorber did not affect the measurement results.

The positron decay of the ^68^Ga was detected in the LS channel, with count rate *N_β_*, while electron capture events from both ^68^Ga and ^68^Ge were avoided by constraining the lower level discriminator (LLD) on the amplified signal to be above about 20 keV beta energy. In this way, the determined activity value was independent of any atomic transitions (all below 11 keV), and directly proportional to the total positron emission probability. The LS positron efficiency, *ε_β_*, was varied between about 0.5 and 0.95 using the LLD and extrapolated to 1.0. The NaI(Tl) detector was gated on the 511 keV region using a single channel analyzer and the total *γ*-ray (*N_γ_*) and anticoincidence (*N_AC_*) count rates were recorded. The extrapolation parameter used was *Y* ≡ *N_AC_* / *N_γ_* ≈ (1 − *ε_β_*). Most of the *γ*-ray counts were due to positron-annihilation decays, detected with efficiency,

$$\varepsilon_{\text{ann}}≅\frac{N_{\gamma }}{N_{0}\left(b_{1}+b_{2}\right)},$$ (1)

where *N*_0_ is the activity and (*b*_1_ + *b*_2_) is the total positron emission probability. There was an additional, approximately 0.2 %, contribution from Compton scattering of 1077 keV *γ*-rays, detected with efficiency *ε_γ_*_1077_. Since some of these 1077 keV *γ*-rays correspond to electron capture events, and not positron emission, a small (0.2 %) correction to the intercept was necessary. The modified extrapolation formula is,

$$N_{\beta }=N_{0}\left(b_{1}+b_{2}\right)\left(1-Y\right)\left(\frac{1+\frac{\varepsilon_{\gamma 1077}}{\varepsilon_{\text{ann}}}\frac{b_{3}}{b_{1}+b_{2}}}{1+\frac{\varepsilon_{\gamma 1077}}{\varepsilon_{\text{ann}}}\frac{b_{2}}{b_{1}+b_{2}}}\right),$$ (2)

where the numerator and denominator of the correction term correspond to total *γ*-rays and anticoincident *γ*-rays, respectively, and the branching probabilities *b*_1_, *b*_2_, and *b*_3_ are illustrated and enumerated in Fig. 1. Note that the extrapolation is linear in *Y*, and the *Y* = 0 (*ε_β_* = 1) intercept is given by,

$$N_{\beta_{\mathrm{int}}}\approx N_{0}\left(b_{1}+b_{2}\right)\left(1+\frac{\varepsilon_{\gamma 1077}}{\varepsilon_{\text{ann}}}\frac{b_{3}-b_{2}}{b_{1}+b_{2}}\right).$$ (3)

Equation (3) is similar to Eq. (4) in [8], with a significant difference that here we account for the fact that some of the 1077 keV *γ*-rays do correspond to a positron branch, and thus do not need to be subtracted. This effect is accommodated by the presence of *b*_2_ in the numerator of the correction term (final term in Eq. (3)). Corrections due to the LS efficiency for *γ*-rays and for coincidences due to those events were not necessary, as described below.

An estimate of *ε_γ_*_1077_ was obtained during a separate set of measurements with a ^60^Co point-source and *ε*_ann_ was estimated using Eq. (1). The correction term was checked by exaggerating the effect during additional measurements made with various *γ*-ray energy gates. Gates *G1*, *G2*, and *G3* corresponded to a gate around the Compton region of the annihilation spectrum, the annihilation photopeak (511 keV) and the annihilation sum peak (1022 keV) respectively. The sum peak was unresolved from the 1077 keV peak in the NaI(Tl) detector events. As designed, the data from gates *G1* and *G3* required large corrections due to reduced *ε*_ann_ and enhanced *ε_γ_*_1077_ respectively. The uncorrected and corrected *N*_0_ values are shown in Table 1. The fact that the large corrections for *G1* and *G3* gave consistent results with *G2*, supports the use of this method for the small (0.2 %) correction to the final *N*_0_ value, based on *G2* alone.

**Fig. 3:**
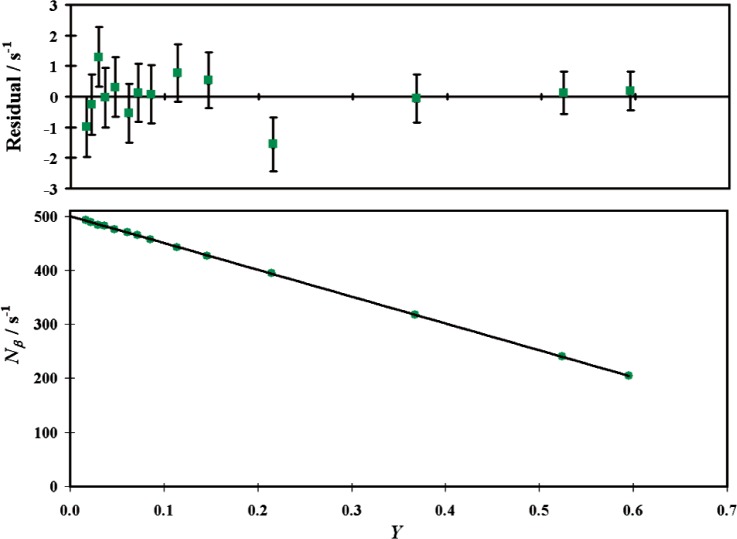
Typical ^68^Ga LS rate, *N_β_*, extrapolation versus anticoincidence efficiency, *Y* (bottom) and residual, *R*, from a linear least-squares fit versus *Y* (top). Statistical (*k* = 1) uncertainties are shown in the residual plot.

No correction in the final result was made for the gamma efficiency of the LS detector, or for coincidences between such events and the NaI(Tl) detector. For gates *G1* and *G2*, these effects tend to cancel out due to the two-photon annihilation process. If one photon is detected in the LS detector, that efficiency can be monitored by the other photon interacting in the NaI detector [15]. If such an effect were present it would lead to a non-linear efficiency extrapolation. A typical *G2* data set and residuals from a linear least-squares fit are shown in Fig. 3 and it is evident that a linear fit is satisfactory. Yet, a quadratic extrapolation was needed to fit the entire range of *G3* (sum peak) data due to the unmonitored LS efficiency for *γ*-rays in that configuration. Thus, for *G3*, a smaller *ε_β_* range (0.9 – 0.95) was employed such that linear and quadratic fits gave consistent results. This value was only used for the *ε_γ_*_1077_ sensitivity test.

Another possible cause for a non-linear extrapolation would be if both the LS and NaI(Tl) efficiencies differed depending on whether the positron was stopped in the LS hemisphere, or escaped before annihilating. This effect was mitigated by three factors: (1) the fact that most positrons annihilated within the hemisphere, (2) the high LS efficiency, and (3) the well-type geometry of the NaI(Tl) detector. The sensitivity of the result to this effect was tested by placing an approximately 0.5 cm thick aluminum foil over the hemisphere and comparing the resulting activity determination. No change in the goodness of the linear fit was detected and the ratio of the intercept with to without the foil was 1.000 ± 0.001, where the uncertainty is a standard (*k* = 1) uncertainty on the linear fit coefficients.

### 2.4 Liquid Scintillation Counting Using the Triple-to-Double Coincidence Ratio (TDCR) Method

Each counting source was counted in the NIST TDCR system [17] on at least two separate occasions over the course of 27 days. Counting times were typically 1200 s, which allowed for the accumulation of at least 10^6^ counts in each of the three doubles counting channels. For each counting experiment, data were acquired at a minimum of 4 efficiency points, which were varied through the use of a set of grey filters that were fitted over the LS vials. Data were acquired in triplicate at each efficiency point. The experimental efficiencies for the logical sum of double photon coincidence events, *ε*_LSD_, ranged from 0.89 to 1.14.

**Fig. 4:**
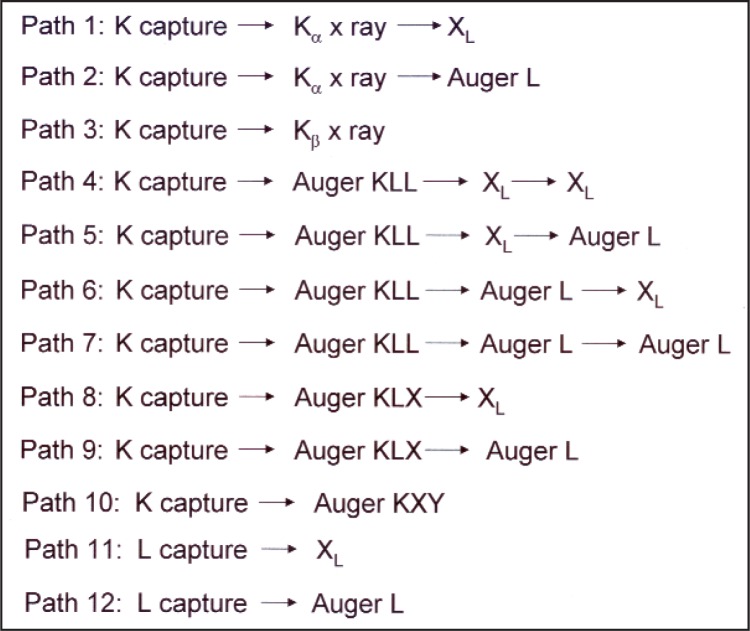
Energy decay schemes used in calculating TDCR efficiencies for electron capture branches in the decay of ^68^Ge and ^68^Ga.

The counting data were analyzed using a program developed in-house for use with the *Mathematica* [18] symbolic mathematics package. Details of the program and the computation strategy will be published separately. However, it should be noted that the program calculates the total detection efficiency for the case of decay of ^68^Ge in equilibrium with its ^68^Ga daughter. To do this, the program was required to solve the TDCR equations [9,10,19,20] for the EC branch of the ^68^Ge parent, as well as both the EC and positron decay of the ^68^Ga daughter. A relatively simplistic model, considering twelve possible decay pathways, was adopted to describe the atomic transitions encountered in the EC decay of ^68^Ge and ^68^Ga. These are depicted in Fig. 4. The values of the various nuclear and atomic input data were taken from the evaluation of the Decay Data Evaluation Project (DDEP) [4].

The analysis program calculates the individual phototube efficiencies, thereby allowing for correction due to asymmetry in the counting rates in each of the-doubles counting channels. The contribution to the detection efficiency due to detection of the 511 keV anihillation photons was taken into account by using the positron spectrum calculated by the program SPEBETA [21] as input for the Monte Carlo simulation package PENELOPE [22] using the techniques described in [23]. The resulting spectrum of energy (positrons+annihilation photons) absorbed in the LS cocktail was then used as input data for the TDCR analysis code.

The stopping power, d*E*/d*x*, for electrons in the LS cocktail was calculated by fitting a function of the form

$$\left(dE/dx\right)=a+bE+c{\left(\ln E\right)}^{2}+d\ln E/E+e/E$$ (4)

(*E* is the value of the midpoint energy for each bin of the calculated beta spectrum and *a*, *b*, *c*, *d*, and *e* are fitting parameters) to data from the NIST ESTAR [24] database using previously published LS cocktail compositions [25].

**Fig. 5:**
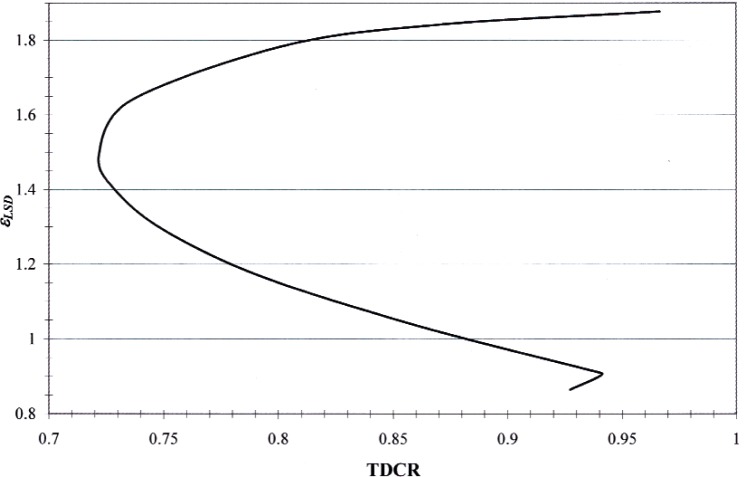
Plot of theoretical efficiency of the logical sum of double coincidence events *ε*_LSD_) as a function of TDCR for ^68^Ge/^68^Ga, assuming equal photomultiplier tube efficiencies and a *kB* value of 0.012 cm · MeV^−1^.

A separate program, assuming equal phototube efficiencies, was developed for evaluating the effects of varying different input and model parameters. Calculations of *ε*_LSD_ were made as a function of the TDCR for *kB* values between 0.009 cm · MeV^−1^ and 0.018 cm · MeV^−1^ and the resulting *ε*_LSD_ values were found to be insensitive to the value of *kB*. For consistency with previous measurements made in this laboratory [26], the value of *kB* for all analyses was taken to be 0.012 cm · MeV^−1^. A plot of the theoretical *ε*_LSD_ values as a function of TDCR at *kB* = 0.012 cm · MeV^−1^ is shown in Fig. 5.

### 2.5 Liquid Scintillation Counting Using the CIEMAT-NIST ^3^H-Standard Efficiency Tracing (CNET) Method

Each LS cocktail was sequentially counted for 10 cycles of 25 min per source in a Packard (Perkin Elmer, Waltham, MA) 2500TR LS spectrometer. Samples were then removed from the counter, agitated and sequentially counted for 10 cycles of 30 min per source in a Beckman LS6500 (Beckman Coulter, Fullerton, CA) spectrometer.

**Fig. 6:**
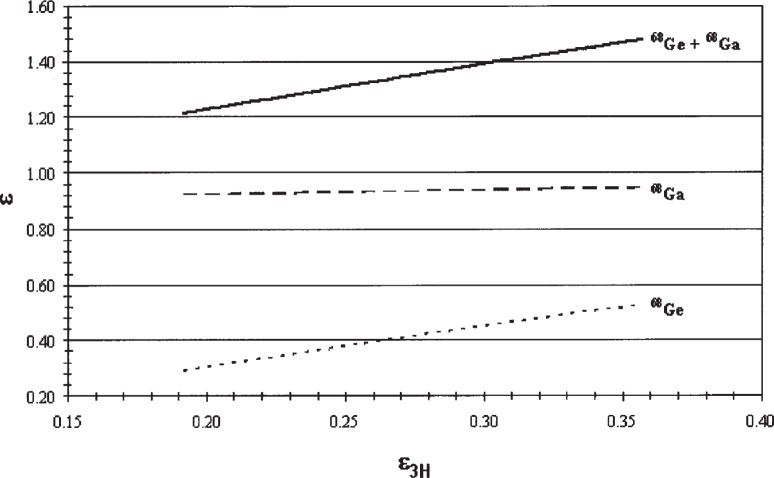
Plot of theoretical ^68^Ge (dotted line), ^68^Ga (dashed line), and total (solid line) efficiencies as a function of tritium efficiency for Ultima Gold, assuming a *kB* value of 0.0075 cm · MeV^−1^.

Efficiency tracing involves calculating a relationship between the measured ^3^H LS efficiencies and the LS efficiencies expected for ^68^Ge, in equilibrium with its daughter ^68^Ga, over a range of experimental quench indicating parameters [7]. The efficiency tracing computer program CN2004 [27] was used in the analysis of the LS data after changing the default input file to include the nuclear and atomic data found in the DDEP evaluation [4]. The average calculated ^68^Ge/^68^Ga efficiency was nominally 138 % in the Packard LS counter and 147 % in the Wallac LS counter using a *kB* value of 0.0075 cm · Mev^−1^ and assuming that the cocktail had the composition of Ultima Gold as specified in the default CN2004 input files. Plots of the calculated theoretical ^68^Ge, ^68^Ga, and total efficiencies as a function of ^3^H tritium are given in Fig. 6.

### 2.6 Ionization Chamber Measurements

For the NIST IC measurements, both *A1* and *A1D1* were measured 40 times each, in four groups of 10 measurements, alternating with 5 groups of 10 measurements of either radium (^226^Ra) reference source RRS100 or RRS500b. Results are analyzed as a ratio of the response of the ampoule to the response of the RRS. After correction for background, the resulting ratio is used to derive a calibration factor, or K-value, defined as the activity of a given radionuclide that would produce the same response as the RRS. The relative values of the RRS100 and RRS500b are well characterized. By determining the K-value using the activity derived from different ampoules of different activity levels, it is also possible to verify the gravimetric dilution factor. The dilution factor from *A1* to *A1D1* was verified by this method to within 0.022 %. The LTAC activity values and the mass dispensed into *A1* were used to determine K-values that can be used for future measurements of ^68^Ge in the NIST ampoule geometry.

### 2.7 Gamma Ray Spectrometry

The solution that remained in *A1D2* after making the LS cocktails was analyzed for possible photon-emitting radionuclidic impurities using two calibrated High-Purity Germanium (HPGe) photon spectrometers at two different counting distances each. In addition, the data provided an additional, confirmatory measurement of the activity concentration using the 1078 keV gamma ray from the decay of ^68^Ga. Characteristics of the detectors used in this study are given in Table 2.

Data were collected using the GammaVision-32 (Ortec, Oak Ridge, TN) software package and analyzed using both GammaVision-32 and Genie 2000 (Canberra, Meriden, CT). Detection efficiencies were calculated from efficiency-energy relationships determined using solutions previously calibrated at NIST and measured in the 5 mL NIST ampoule geometry.

## 3. Results and Discussion

### 3.1 Impurity Analyses

No photon-emitting radionuclidic impurities were detected in solution *A1* to within the following limits (at the reference time) of the massic photon emission rate:

$$\begin{array}{l} 785s^{-1}\cdot g^{-1}\;\text{for}\;30\text{keV}\leq E\geq 507\text{keV};\;\text{and}\\ 285s^{-1}\cdot g^{-1}\;\text{for}\;515\text{keV}\leq E\geq 1800\text{keV}; \end{array}$$

where *E* is the gamma-ray energy.

### 3.2 Activity Measurements Results

The results of the massic activity determinations for the solution contained in *A1* as of the reference time are given in Table 3. The values in Table 3 take into account the dilution factor of 204.903231 between the solution in *A1* and that used in the assays, *A1D2*. The uncertainties given in the table are expanded (*k* = 2) uncertainties based on the components given in Tables 4–7.

Of the different techniques used in this study to determine the activity concentration of the ^68^Ge solution, the LTAC technique is much less dependent on level scheme data and model parameters not directly measured in the experiment. For this particular measurement, the only input parameter significantly impacting the activity calculation that was not directly measured in the experiment was the positron branching ratio. The other branching ratios only contributed to the minor (0.2 %) correction for the leakage of 1077 keV *γ*−rays into the annihilation *γ*-ray gate. And even this small correction was checked experimentally by modifying the experimental design to exaggerate the effect and then verifying that the corrected activity agreed with the original value.

On the other hand, our implementations of the TDCR and CNET efficiency tracing methods are unable to separate the positron and EC decay signals and must therefore account for all possible decay paths, including atomic rearrangements following electron capture. From a practical standpoint, a compromise between treating all possible paths and reasonable computation times must be made. While this certainly introduces some small amount of uncertainty, it is not expected that the weak contributions due to paths not considered in Fig. 4 would be significant, at least for the TDCR method. Instead, as seen in Table 5, the uncertainties on the input data play a very significant role.

Because of the more direct nature of the measurement in the LTAC technique, the LTAC activity value for the solution in *A1* was adopted as the reference value for this study and was used in the calculation of the K-value for the NIST IC. The fact that the LTAC and TDCR measurements agree to within their respective experimental uncertainties is encouraging, given the complexity of the TDCR efficiency calculation. Nonetheless, one would hope that improvements in the NIST TDCR spectrometer would lead to higher EC detection efficiencies, thereby providing better results in the measurement of radionuclides that decay by this mode. The CNET results indicate that some improvements in the method are still needed to be able to reliably measure nuclides that undergo EC decay.

### 3.3 Determination of K-Value for NIST IC

In order to avoid the need to perform a primary standardization every time a NIST-calibrated solution of ^68^Ge/^68^Ga is required, we determined a calibration factor (K-value) for the NIST IC. This K-value is not to be confused with the coverage factor, *k*, applied to uncertainty evaluations. Using the LTAC reference activity value and the measured responses in the IC against radium reference sources (RR) 500B and 100, the K-values were found to be 2.695 × 10^7^ ± 1.7 × 10^5^ Bq and 5.032 × 10^6^ ± 3.2 × 10^4^ Bq, respectively. The uncertainties on the K-values are expanded (*k* = 2) uncertainty and include relative standard uncertainty components due to the original primary standardization (0.29 %), repeatability on 40 measurements in the IC (0.015 %), source mass (0.05 %), decay correction (0.002 %), and source positioning (0.1 %).

## 4. Conclusion

A solution containing ^68^Ge in equilibrium with its decay daughter ^68^Ga has been standardized for the first time at NIST, with a combined standard uncertainty of 0.29 % using LTAC. Measurements made with two other LS techniques, TDCR and CNET, confirmed the LTAC result to within 1.1 % and 1.5 %, respectively. The differences between results obtained with the latter two methods and the LTAC technique indicate that improvements in the models and/or their applications are needed, particularly for EC nuclides.

Data collected on the NIST 4π*γ* ionization chamber allowed for the determination of calibration factors for that chamber in the 5 mL NIST ampoule geometry, thereby enabling future calibrations of solutions having the same solution composition without the need for the measurements to be made by a primary method.

## Figures and Tables

**Table 1:** Calculated ^68^Ga activity from various *γ*-ray gates and using Eq. (3), with and without the final correction term, and relative to the corrected *G2* value, (used for the final activity determination). The uncertainties (*k* = 1) on the uncorrected activities are standard deviations of the intercepts from the least-squares fits to the various data sets. The uncertainties (*k* = 1) of the corrected values are from estimates of the uncertainties in *ε*_ann_ and *ε_γ_*_1077_

*γ*-ray gate	Uncorrected *N*_0_ (Bq)	Corrected *N*_0_ (Bq)
*G1* - Comptons	1.020 ± 0.002	1.002 ± 0.004
*G2* - photopeak	1.0021 ± 0.001	1.0000 ± 0.0004
*G3* - sum peak	1.033 ± 0.002	1.000 ± 0.007

**Table 2:** Characteristics of HPGe detectors used in the present study

Detector parameter	X-detector	B-detector
Detector diameter	43.6 ± 0.1 mm	54.9 ± 0.1 mm
Detector length	36.2 ± 0.1 mm	54.2 ± 0.05 mm
End cap window material	Beryllium	Beryllium
Window thickness	0.5 ± 0.05 mm	0.5 ± 0.05 mm
Crystal-window distance	3 ± 0.5 mm	3 ± 0.5 mm
Crystal top dead zone thickness	0.3 ± 0.03 μm	0.3 ± 0.03 μm
Crystal material	Germanium	Germanium
Crystal hole depth	32.6 ± 0.5 mm	47.2 ± 0.5 mm
Crystal hole diameter	10.4 ± 0.5 mm	12 ± 0.5 mm
Detector side cap thickness	1.3 ± 0.1 mm	1.3 ± 0.1 mm
Detector side cap diameter	70 ± 1 mm	63.5 ± 0.5 mm
Detector side cap material	Aluminum	Magnesium
Detector type	*n*-type	*n*-type
Calibration Geometries (distances are source-to-detector)	Ampoule; side-mount, end-on 24 cm	Ampoule; 24 cm, 35 cm

**Table 3:** Results of massic activity determinations (C_A_, in Bq · g^−1^) for the ^68^Ge solution contained in ampoule *A1* as of the reference time of 12:00 EST 1 May 2007. The uncertainties, given in parentheses, are expanded (*k* = 2) uncertainties based on the evaluated uncertainty components listed in Tables 4–7 for the respective techniques

Technique	*C*_A_, 10^6^ Bq · g^−1^
4π*β* - *γ* anticoincidence counting (LTAC)	3.104(18)
LS counting with the Triple-to-Double Ratio (TDCR) method	3.141(25)
LS counting with the CIEMAT/NIST ^3^H-standard efficiencytracing method (CNET)	3.058(44)
Gamma-ray spectrophotometry with High Purity Germanium (HPGe) detectors	3.2(9)

**Table 4:** Uncertainty components evaluated in the determination of the massic activity, *C*_A_, for ^68^Ge solution *A1* by 4π*β* - *γ* anticoincidence counting (LTAC)

Component, *u_i_*	Comment	Evaluation type	%
Measurement variability	Standard deviation of the mean on the determination of *C*_A_ for 8 trials encompassing 4 samples, 3 backgrounds, 2 extending dead-times and absorber/no absorber- over a 2-week period	A	0.03
Background variability	Additional variation estimate based on 3 trials and within-run variation	A	0.03
Additional variability in background	Additional variability not embodied by “random sources”	B	0.05
Half-life	Standard uncertainty in half-life. (0.059 %) over the measurement decay interval	B	0.0008
Livetime	Estimated standard uncertainty on *C*_A_ due to uncertainty in counting livetime.	B	0.1
Branching ratio	Estimated standard uncertainty due to uncertainty in published decay branching ratios	B	0.13
Extrapolation	Estimated standard uncertainty due to extrapolation to zero non-detection efficiency; based on sensitivity tests, previous measurements and models	B	0.2
Correction due to detection of 1077 keV photons	Estimated standard uncertainty on *C*_A_ due to efficiency of detecting 1077 keV photons based on sensitivity tests	B	0.1
Mass determinations	Estimated standard uncertainty of mass for any single LS cocktail	B	0.05
Dilution factor	Uncertainty in *C*_A_ of solution Ge1A1 due to uncertainty in gravimetrically-determined dilution factor between solutions in ampoules Ge1A1 and Ge1A1D2	B	0.04

Combined $\left(u_{c}=\surd \sum u_{i}^{2}\right)$			0.29

Expanded (*U*_c_ = *u*_c_ · *k*; *k* = 2)			0.58

**Table 5:** Uncertainty components evaluated in the determination of the massic activity, *C*_A_, for ^68^Ge solution *A1* by liquid scintillation counting using TDCR method

Component, *u_i_*	Comment	Evaluation type	%
Sample repeatability	Standard deviation of the mean on the determination of massic activity for a single LS cocktail (*n* = 3 – 5 determinations of *C*_A_ per source)	A	0.06
LS cocktail composition variability	Standard deviation on the determination of *C*_A_ for three LS cocktail compositions (*n* = 6 – 19 determinations of *C*_A_ per composition)	A	0.09
Efficiency dependence	Median difference between maximum and minimum value of *C*_A_ determined for a single source at between 3 and 4 efficiency values, varied by use of grey filters (*n* = 12 independent measurements)	B	0.20
Effect of ^68^Ga beta endpoint energy, E*_β,_*_max_ on efficiency calculations	Standard uncertainty in efficiency calculation due to standard uncertainties on positron endpoint energies of ^68^Ga	B	0.18
Effect of other atomic and nuclear input data	Standard uncertainty due to uncertainties on data used as input to the TDCR analysis code as determined by Monte Carlo methods. A total of 20 data sets were generated from normal distributions defined by the published nuclear and atomic data and their associated standard uncertainties, which were taken as the standard deviation of the respective distributions. Each data set was used to calculate *C*_A_ using a single experimental data set	A	0.26
Half-life	Standard uncertainty in half-life (0.059 %) over the measurement decay interval	B	6·10^−3^
Mass determinations	Estimated standard uncertainty of mass for any single LS cocktail	B	0.05
Livetime	Standard uncertainty arising from an estimated uncertainty of 0.007 % on the determination of the live time	B	7 · 10^−3^
Background	Standard deviation on the determination of *C*_A_ determined via Monte Carlo simulation. A total of 5 background data sets were constructed from random data arising from normal distributions defined by the average and standard deviation of experimental backgrounds at 4 efficiency points having 3 repetitions each; calculations were carried out with all 5 background data sets for a single experimental data set	A	5 · 10^−3^
Dilution factor	Uncertainty in *C*_A_ of solution Ge1A1 due to uncertainty in gravimetrically-determined dilution factor between solutions in ampoules Ge1A1 and Ge1A1D2	B	0.04

Combined $\left(u_{c}=\surd \sum u_{i}^{2}\right)$			0.39

Expanded (*U*_c_ = *u*_c_ · *k*; *k* = 2)			0.79

**Table 6:** Uncertainty components evaluated in the determination of the massic activity, *C*_A_, for ^68^Ge solution *A1* by liquid scintillation counting using CIEMAT/NIST 3H standard efficiency tracing method

Component, *u_i_*	Comment	Evaluation type	%
Sample repeatability	Standard deviation of the mean on the determination of massic activity for a single LS cocktail (*n* = 10 determinations of *C*_A_ per source)	A	0.05
LS measurement reproducibility	Standard deviation on the determination of *C*_A_ for 10 cocktails of 2 compositions	A	0.24
Mass determinations	Estimated standard uncertainty of ^68^Ge mass for any single LS cocktail	B	0.05
Dilution factor	Uncertainty in *C*_A_ of solution Ge1A1 due to uncertainty in gravimetrically-determined dilution factor between solutions in ampoules Ge1A1 and Ge1A1D2	B	0.04
^68^Ge decay corrections	Standard uncertainty in half-life (0.059 %) over the measurement decay interval	B	0.001
^68^Ge efficiency	Estimated uncertainty in *C*_A_ due to step size in CN2004 calculations	B	0.65
Livetime determinations	Estimated uncertainty in the correction to the LS counting interval	B	0.05 (and PE)^3^
Background	Estimated uncertainty due to an average 4 % uncertainty in background determination	B	0.004
Activity of 3H standard	Estimated uncertainty due to 0.36 % uncertainty in 3H standard activity	B	0.18
Branching ratios	Estimated uncertainty due to uncertainty in branching ratios	B	0.08

Combined $\left(u_{c}=\surd \sum u_{i}^{2}\right)$			0.73

Expanded (*U*_c_ = *u*_c_ · *k*; *k* = 2)			1.45

3 The relative uncertainty for this component is partially embodied (PE) in the relative standard uncertainties of the repeatability and reproducibility components.

**Table 7:** Uncertainty components evaluated in the determination of the massic activity, *C*_A_, for ^68^Ge solution Ge1A1 by *γ*-ray spectrometry using HPGe detectors

Component, *u_i_*	Comment	Evaluation type	%
Measurement repeatability	Standard deviation on determination of *C*_A_ for 3 repeated measurements of a single source at a single geometry	A	0.98
Efficiency curve	Standard deviation of the mean on determination of detection efficiency for 4 sample geometries	B	0.12
Sample geometry	Typical uncertainty due to change of sample geometry (detector and source-to-detector distance) for a single counting source	B	0.33
Decay correction	Standard uncertainty in half-life (0.059 %) over the measurement decay interval	B	2.6 · 10^−3^
Decay data	Standard uncertainty (0.93 %) on emission probablity of 1078 keV gamma-ray in the decay of ^68^Ge	B	0.93
Dilution factor	Estimated standard uncertainty in *C*_A_ of solution Ge1A1 due to uncertainty in gravimetrically-determined dilution factor between solutions in ampoules Ge1A1 and Ge1A1D2	B	0.04

Combined $\left(u_{c}=\surd \sum u_{i}^{2}\right)$			1.4

Expanded (*U*_c_ = *u*_c_ · *k*; *k* = 2)			2.8

## Footnotes

2 Certain commercial equipment, instruments, or materials are identified in this paper to foster understanding. Such identification does not imply recommendation or endorsement by the National Institute of Standards and Technology, nor does it imply that the materials or equipment identified are necessarily the best available for the purpose.
